# Left ventricular mass predicts cardiac reverse remodelling in patients treated with empagliflozin

**DOI:** 10.1186/s12933-023-01849-w

**Published:** 2023-06-28

**Authors:** Pankaj Puar, Makoto Hibino, C. David Mazer, Andrew T. Yan, Arjun K. Pandey, Adrian Quan, Hwee Teoh, David A. Hess, Raj Verma, Kim A. Connelly, Subodh Verma

**Affiliations:** 1 Division of Cardiac Surgery, St. Michael’s Hospital of Unity Health Toronto, 30 Bond Street, Toronto, ON Canada; 2 Keenan Research Centre for Biomedical Science in the Li Ka Shing Knowledge Institute of St. Michael’s Hospital, Toronto, ON Canada; 3 Department of Pharmacology and Toxicology, University of Toronto, Toronto, ON Canada; 4 Faculty of Medicine, University of British Columbia, Vancouver, BC Canada; 5 Department of Cardiac Surgery, University Hospitals Cleveland Medical Center, Case Western Reserve University, Cleveland, OH USA; 6 Department of Anesthesia, St. Michael’s Hospital of Unity Health Toronto, Toronto, ON Canada; 7 Departments of Anesthesiology and Pain Medicine, University of Toronto, Toronto, ON Canada; 8 Department of Physiology, University of Toronto, Toronto, ON Canada; 9 Division of Cardiology, St. Michael’s Hospital of Unity Health Toronto, Toronto, ON Canada; 10 Department of Medicine, University of Toronto, Toronto, ON Canada; 11 Michael G. DeGroote School of Medicine, McMaster University, Hamilton, ON Canada; 12 Division of Endocrinology and Metabolism, St. Michael’s Hospital of Unity Health Toronto, Toronto, ON Canada; 13 Molecular Medicine Research Laboratories, Krembil Centre for Stem Cells Biology, Robarts Research Institute, University of Western Ontario, London, ON Canada; 14 Department of Physiology and Pharmacology, University of Western Ontario, London, ON Canada; 15 Royal College of Surgeons in Ireland, Dublin, Ireland; 16 Department of Surgery, University of Toronto, Toronto, ON Canada

**Keywords:** SGLT2 inhibition, Diabetes, Heart failure, Cardiac reverse remodelling, Left ventricle

## Abstract

**Background:**

The cardiovascular (CV) benefits of sodium-glucose transport protein 2 inhibitors have been attributed, in part, to cardiac reverse remodelling. The EMPA-HEART CardioLink-6 study reported that sodium-glucose cotransporter-2 inhibition for 6 months with empagliflozin was associated with a significant reduction in left ventricular mass indexed to body surface area (LVMi). In this sub-analysis, we evaluated whether baseline LVMi may influence how empagliflozin affects cardiac reverse remodelling.

**Methods:**

A total of 97 patients with type 2 diabetes and coronary artery disease were randomized to empagliflozin (10 mg/d) or matching placebo for 6 months. The study cohort was divided into those whose baseline LVMi was ≤ 60 g/m^2^ and those who had a baseline LVMi > 60 g/m^2^. Subgroup comparisons were conducted using a linear regression model adjusted for baseline values (ANCOVA) that included an interaction term between LVMi subgroup and treatment.

**Results:**

Baseline LVMi was 53.3 g/m^2^ (49.2–57.2) and 69.7 g/m^2^ (64.2–76.1) for those with baseline ≤ 60 g/m^2^ (n = 54) and LVMi > 60 g/m^2^ (n = 43) respectively. The adjusted difference of LVMi regression between those randomized to empagliflozin and placebo were − 0.46 g/m^2^ (95% CI: −3.44, 2.52, *p* = 0.76) in the baseline LVMi ≤ 60 g/m^2^ subgroup and − 7.26 g/m^2^ (95% CI: −11.40, −3.12, *p* = 0.0011) in the baseline LVMi > 60 g/m^2^ subgroup (*p*-for-interaction = 0.007). No significant associations were found between baseline LVMi and 6-month change in LV end systolic volume-indexed (*p*-for-interaction = 0.086), LV end diastolic volume-indexed (*p*-for-interaction = 0.34), or LV ejection fraction (*p*-for-interaction = 0.15).

**Conclusions:**

Patients with higher LVMi at baseline experienced greater LVM regression with empagliflozin.

## Background

Sodium-glucose transport protein 2 inhibitors (SGLT2i) have shown marked cardiovascular and renal benefits in patients with type 2 diabetes (T2D) [1–5]. More specifically, the Cardiovascular Outcome Event Trial in Type 2 Diabetes Mellitus Patients (EMPA-REG OUTCOME) showed that the SGLT2i, empagliflozin, significantly reduced the occurrence of all-cause mortality, cardiovascular-related mortality, and hospitalization for heart failure in patients with T2D and cardiovascular disease [2]. More recent trials have suggested benefits of SGLT2i in the treatment of both heart failure with reduced and preserved ejection fraction, and in people with and without T2D [6–9].

There have been many suggested mechanisms to explain the cardiovascular benefits associated with SGLT2i [10–15], one of which is cardiac reverse remodelling. In support of this suggestion, a recent meta-analysis of randomized controlled trials (RCTs) reported that treatment with SGLT2i was associated with significant reductions in left ventricular mass (LVM) as detected by cardiac magnetic resonance imaging (cMRI) [9]. More specifically, the EMPA-HEART CardioLink-6 (Effects of Empagliflozin on Cardiac Structure in Patients with Type 2 Diabetes) randomized controlled trial of 97 patients with T2D and coronary artery disease found that treatment with empagliflozin significantly reduced left ventricular mass indexed to body surface area (LVMi) over 6 months [16]. These results support the notion that the clinical benefits provided by SGLT2i may be explained, in part, through left ventricular (LV) reverse remodelling.

Left ventricular hypertrophy (LVH) is an established predictor of poor cardiovascular outcomes, while increases in LVMi have been independently associated with all-cause mortality and sudden death [17–23]. In this exploratory sub-analysis of the EMPA-HEART CardioLink-6 trial, we evaluated the influence of baseline LVMi on cardiac reverse remodelling following 6 months of treatment with empagliflozin.

## Methods

A detailed description of the design and primary results of the EMPA-HEART CardioLink-6 trial has been published previously [16]. In brief, 97 patients between the ages of 40 and 80 years old with T2D and coronary artery disease were randomized to either empagliflozin (10 mg/d) or matching placebo for 6 months. Cardiac parameters were measured at baseline and end-of-study by cMRI according to a standardized protocol with blinded image analysis that has been described in detail elsewhere [16].

Given that the median baseline LVMi for the empagliflozin-assigned group was 58 kg/m^2^ and 60 kg/m^2^ for the placebo-allocated group [16], for this sub-analysis, the cohort was stratified into those with a baseline LVMi ≤ 60 g/m^2^ (N = 54) and those with a baseline LVMi > 60 g/m^2^ (N = 43). Our primary analysis evaluated the change in LVMi from baseline to 6 months after treatment with empagliflozin in each of the LVMi ≤ 60 g/m^2^ and LVMi > 60 g/m^2^ subgroups. We also evaluated the association between baseline LVMi as a continuous variable and change in LVM from baseline to 6-months.

Our secondary analyses included assessment of the relationship between baseline LVMi and changes in left ventricular end-systolic volume indexed to body surface area (LVESVi), left ventricular end-diastolic volume indexed to body surface area (LVEDVi), and left ventricular ejection fraction (LVEF) from baseline to 6-months. We also tested the associations between baseline LVMi and baseline left ventricular end-systolic volume (LVESV), LVESVi, left ventricular end-diastolic volume (LVEDV), LVEDVi, and LVEF.

### Statistical analyses

Normality of continuous variables was tested with the Skewness and Kurtosis test and examined with visual inspection of a histogram. Continuous variables are reported as medians with interquartile ranges (IQR) or mean ± standard deviation (SD); frequencies and percentages are used to describe categorical data. Continuous variables were assessed with the Mann-Whitney U-test. Categorical variables were evaluated with the Pearson’s chi-square test (or Fisher’s exact test if appropriate). To assess the treatment effect on 6-month change in LVMi in each of the LVMi stratified sub-groups we used a linear model adjusting for baseline differences in LVMi (ANCOVA), that included an interaction term between the baseline LVMi subgroup and treatment. We also estimated treatment effect over baseline LVMi values from 40 to 90 g/m^2^ in the ANCOVA. As a sensitivity analysis, we conducted an additional ANCOVA including adjustment for baseline characteristics which showed significant difference between the subgroup divided by LVMi of 60 g/m^2^. We also conducted an ANCOVA for 6-month change in LVESVi, LVEDVi, and LVEF that included adjustment for their baseline values. Treatment effects on 6-month change in each variable between the LVMi stratified sub-groups were assessed using ANCOVA models which include an interaction term between the LVMi subgroup and treatment. The results of the regression models were summarized as adjusted differences with two-sided 95% confidence intervals. We predicted 6-month changes in LVMi from estimation of a fractional polynomial of baseline LVMi and 95% CIs. A *p* < 0.05 was considered statistically significant. All statistical analyses were performed using the STATA statistical software version 17 (StataCorp LP, College Station, TX, USA).

## Results

### Baseline characteristics

Upon stratification of the EMPA-HEART cohort, most baseline characteristics were found to be similar between patients with an LVMi ≤ 60 g/m^2^ and those patients with an LVMi > 60 g/m^2^. A total of 90.7% of patients in the LVMi ≤ 60 g/m^2^ group and 95.3% of patients in the LVMi > 60 g/m^2^ group were male (Table 1). Patients whose baseline LVMi was ≤ 60 g/m^2^ had longer durations of diabetes (10.3 years; 8.0–17.0) than those with baseline LVMi > 60 g/m^2^ (5.5 years; 2.2–15.0) (*p* = 0.002) (Table 1). Patients in the LVMi > 60 g/m^2^ subgroup had significantly higher glucose (random) than patients with LVMi ≤ 60 g/m^2^ (10.1 mmol/L (IQR: 7.0-14.4) vs. 7.6 mmol/L (6.3–10.5) respectively; *p* = 0.013). Additionally, a greater number of patients with LVMi ≤ 60 g/m^2^ were found to have history of prior percutaneous coronary intervention (*p* = 0.043) (Table 1). The median LVMi was 69.7 g/m^2^ (64.2–76.1) in those with an LVMi > 60 g/m^2^ and 53.3 g/m^2^ (49.2–57.2) in those with an LVMi ≤ 60 g/m^2^ (Table 2).

**Table 1:** Baseline characteristics of the EMPA-HEART CardioLink-6 participants as stratified by baseline LVMi of ≤ or > 60 g/m^2^

	LVMi ≤ 60 g/m^2^			LVMi > 60 g/m^2^			*p*-value (LVMi ≤ 60 g/m^2^ vs. LVMi > 60 g/m^2^)
	All N = 54	Empagliflozin N = 30	Placebo N = 24	All N = 43	Empagliflozin N = 19	Placebo N = 24	
Age, years	66 (57–71)	66 (58–71)	67 (57–72)	63 (53–67)	62 (53–65)	64 (52–70)	0.070
Female	5 (9.3%)	3 (10.0%)	2 (8.3%)	2 (4.7%)	2 (10.5%)	0 (0.0%)	NA
BMI, kg/m^2^	26.1 (23.8–29.8)	26.8 (24.2–31.0)	25.5 (23.3–27.6)	27.7 (25.4–30.1)	26.1 (24.7–29.9)	28.4 (26.2–30.2)	0.099
Duration of Diabetes, years	10.3 (8.0–17.0)	11.7 (9.0–17.0)	10.0 (7.0–17.0)	5.5 (2.2–15.0)	4.0 (2.0–15.0)	7.0 (3.0–15.0)	0.002
HbA1c, %	8.0 (7.2–8.3)	8.1 (7.7–8.4)	7.6 (7.2–8.2)	7.9 (7.4–8.7)	7.6 (7.2-8.0)	8.5 (7.4–9.1)	0.54
Glucose (random), mmol/L	7.6 (6.3–10.5)	7.3 (6.6–11.5)	7.8 (6.1–10.2)	10.1 (7.0-14.4)	9.0 (6.8–14.4)	10.5 (7.8–14.5)	0.013
Systolic Blood Pressure, mmHg	130 (120–143)	128 (121–140)	134 (119–144)	134 (121–153)	133 (118–160)	135 (128–149)	0.12
Diastolic Blood Pressure, mmHg	75 (68–81)	74 (68–80)	76 (70–84)	76 (70–82)	75 (68–91)	77 (71–80)	0.65
Cholesterol (random), mg/dL	122.6 (109.4-135.3)	124.1 (114.8-143.5)	118.1 (105.4-130.5)	122.4 (106.7-143.1)	120.3 (101.7-137.3)	123.0 (106.7-149.3)	0.81
LDL-Cholesterol, mg/dL	47.6 (40.0-65.5)	53.8 (41.4–71.9)	44.9 (39.1–59.6)	54.5 (38.7–73.5)	55.3 (39.8–71.2)	53.8 (37.9–78.5)	0.43
HDL-Cholesterol, mg/dL	40.2 (32.1–45.2)	40.2 (32.1–43.7)	38.7 (33.1–46.6)	37.5 (34.8–43.7)	37.1 (35.6–40.2)	37.5 (32.5–46.0)	0.89
Triglyceride, mg/dL	168.3 (119.6-217.9)	170.1 (124.9-221.4)	154.6 (108.9-186.4)	157.2 (110.7-193.1)	164.7 (110.7-201.9)	155.9 (109.8-184.2)	0.82
eGFR, mL/min per 1.73m^2^	87.9 (71.4–97.8)	86.9 (77.7–97.8)	88.4 (67.1–98.6)	86.6 (79.7-100.4)	86.5 (80.4–98.2)	87.6 (79.6-101.9)	0.47
Creatinine, mg/dL	0.9 (0.8-1.0)	0.9 (0.8-1.0)	0.9 (0.9–1.1)	0.9 (0.8-1.0)	0.9 (0.8-1.0)	0.9 (0.8-1.0)	0.68
Hemoglobin, g/dL	13.8 (12.7–15.0)	14.1 (13.1–15.0)	13.4 (12.7–14.8)	14.2 (13.3–15.0)	14.4 (12.8–15.3)	14.1 (13.4–15.0)	0.45
Hematocrit. %	0.42 (0.39–0.44)	0.42 (0.40–0.46)	0.41 (0.39–0.43)	0.43 (0.40–0.44)	0.43 (0.40–0.46)	0.42 (0.40–0.44)	0.32
NT-proBNP, pg/mL	102 (53–178)	93 (44–158)	114 (59–217)	115 (55–329)	107 (58–335)	116 (55–312)	0.15
Previous PCI	30 (55.6%)	19 (63.3%)	11 (45.8%)	15 (34.9%)	7 (36.8%)	8 (33.3%)	0.043
Previous CABG	28 (51.9%)	15 (50.0%)	13 (54.2%)	27 (62.8%)	13 (68.4%)	14 (58.3%)	0.28
Heart Failure	5 (9.3%)	2 (6.7%)	3 (12.5%)	1 (2.4%)	0 (0.0%)	1 (4.2%)	0.23
Hypertension	49 (90.7%)	27 (90.0%)	22 (91.7%)	38 (90.5%)	18 (94.7%)	21 (87.5%)	1.00
Diabetic Nephropathy	2 (3.7%)	0 (0.0%)	2 (8.3%)	0 (0.0%)	0 (0.0%)	0 (0.0%)	0.50
Stroke or TIA	44 (81.5%)	7 (23.3%)	3 (12.5%)	39 (90.7%)	1 (5.3%)	3 (12.5%)	0.25
Peripheral Artery Disease	50 (92.6%)	2 (6.7%)	2 (8.3%)	42 (97.7%)	0 (0.0%)	1 (4.2%)	0.38
Past or Current Smoker	33 (61.1%)	12 (40.0%)	9 (37.5%)	22 (51.2%)	8 (42.1%)	13 (54.2%)	0.33
Metformin	52 (96.3%)	30 (100.0%)	22 (91.7%)	39 (90.7%)	17 (89.5%)	22 (91.7%)	0.40
Insulin	10 (18.5%)	6 (20.0%)	4 (16.7%)	14 (32.6%)	6 (31.6%)	8 (33.3%)	0.11
Statin	51 (94.4%)	29 (96.7%)	22 (91.7%)	42 (97.7%)	18 (94.7%)	24 (100.0%)	0.63
ACEi/ARB	47 (87.0%)	26 (86.7%)	21 (87.5%)	34 (79.1%)	14 (73.7%)	20 (83.3%)	0.41
Furosemide/Thiazide	5 (9.3%)	2 (6.7%)	3 (12.5%)	10 (23.3%)	2 (10.5%)	8 (33.3%)	0.089
Beta Blocker	42 (77.8%)	23 (76.7%)	19 (79.2%)	34 (81.0%)	15 (78.9%)	20 (83.3%)	0.80
Calcium Channel Blocker	11 (20.4%)	3 (10.0%)	8 (33.3%)	10 (23.3%)	3 (15.8%)	7 (29.2%)	0.81
ASA/P2Y12 Inhibitor	43 (79.6%)	23 (76.7%)	20 (83.3%)	38 (88.4%)	17 (89.5%)	21 (87.5%)	0.28

Data are presented as either frequency (%) or median (IQR).

ACEi, angiotensin-converting enzyme inhibitor; ARB, angiotensin-receptor blocker; ASA, acetylsalicylic acid; CABG, coronary artery bypass graft; eGFR, estimated glomerular filtration rate; HDL, high-density lipoprotein; IGFBP7, Insulin-like growth factor binding protein 7; LDL, low-density lipoprotein; LVMi, left ventricular mass index; NT-proBNP, N-terminal pro-b-type natriuretic peptide; PCI, percutaneous coronary intervention; TIA; transient ischemic attack.

**Table 2:** Baseline cMRI parameters of the EMPA-HEART CardioLink-6 participants stratified by baseline LVMi of ≤ or > 60 g/m^2^

	LVMi ≤ 60 g/m^2^ (N = 54)	LVMi > 60 g/m^2^ (N = 43)	*p*-value
Baseline LVMi, g/m^2^	53.3 (49.2–57.2)	69.7 (64.2–76.1)	< 0.001
Baseline LVM, g	100.2 (88.0–108.5)	137.8 (127.2–162.3)	< 0.001
Baseline LVEDV, mL	118.0 (102.1–139.8)	135.1 (119.2–160.9)	0.010
Baseline LVESV, mL	50.7 (38.2–61.2)	55.7 (44.9–74.5)	0.079
Baseline LVEDVi, mL/m^2^	64.4 (55.2–73.6)	70.8 (60.9–79.8)	0.058
Baseline LVESVi, mL/m^2^	27.7 (21.4–31.7)	26.9 (22.6–39.1)	0.25
Baseline LVEF, %	58.2 (52.2–63.6)	58.3 (49.5–64.0)	0.88

*All data are presented as median (IQR). LVMi, left ventricular mass index; LVM, left ventricular mass; LVEDV, left ventricular end diastolic volume; LVESV, left ventricular end systolic volume; LVEDVi; left ventricular end diastolic volume indexed; LVESVi, left ventricular end systolic volume indexed; LVEF, left ventricular ejection fraction.

### Primary outcomes

**Fig. 1:**
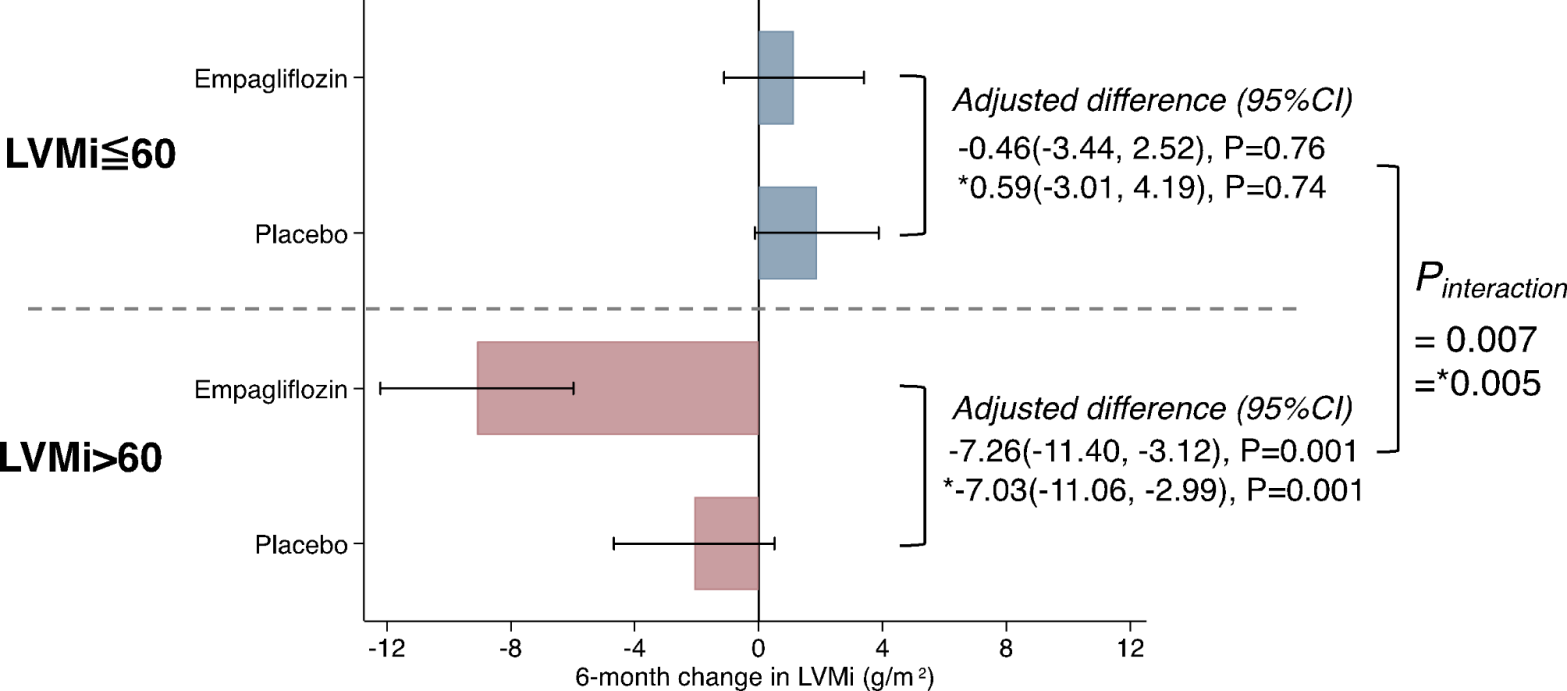
Treatment with empagliflozin (10 mg/d) and 6-month change in LVMi stratified by baseline LVMi of ≤ 60 g/m^2^ or > 60 g/m^2^

The effect of empagliflozin on LVMi regression over 6 months was significantly different between patients with a baseline LVMi ≤ 60 g/m^2^ and those whose LVMi was > 60 g/m^2^ (Fig. 1). The LVMi regression over 6 months in those randomized to empagliflozin and those assigned to placebo was − 0.46 g/m^2^ (95% CI: −3.44, 2.52; *p* = 0.76) and − 7.26 g/m^2^ (95% CI: −11.40, −3.12; *p* = 0.0011) in the LVMi ≤ 60 g/m^2^ and LVMi > 60 g/m^2^ subgroups, respectively (*p*-for-interaction = 0.007). We also conducted an ANCOVA analysis that considered the duration of T2D, random glucose levels, as well as the presence or absence of a previous history of PCI and found a significant difference between the LVMi-stratified groups. These associations persisted after adjustment for these baseline characteristics with adjusted differences of 0.59 g/m^2^ (95% CI: −3.01, 4.19; *p* = 0.74) in the LVMi ≤ 60 g/m^2^ group and − 7.03 g/m^2^ (95% CI: −11.06, −2.99; *p* = 0.001) in the LVMi 60 > g/m^2^ group (*p*-for-interaction = 0.005).

*ANCOVA model adjusted for duration of type 2 diabetes mellitus, glucose (random), previous history of PCI in addition to baseline LVMi. LVMi, left ventricular mass indexed to body surface area.

**Fig. 2:**
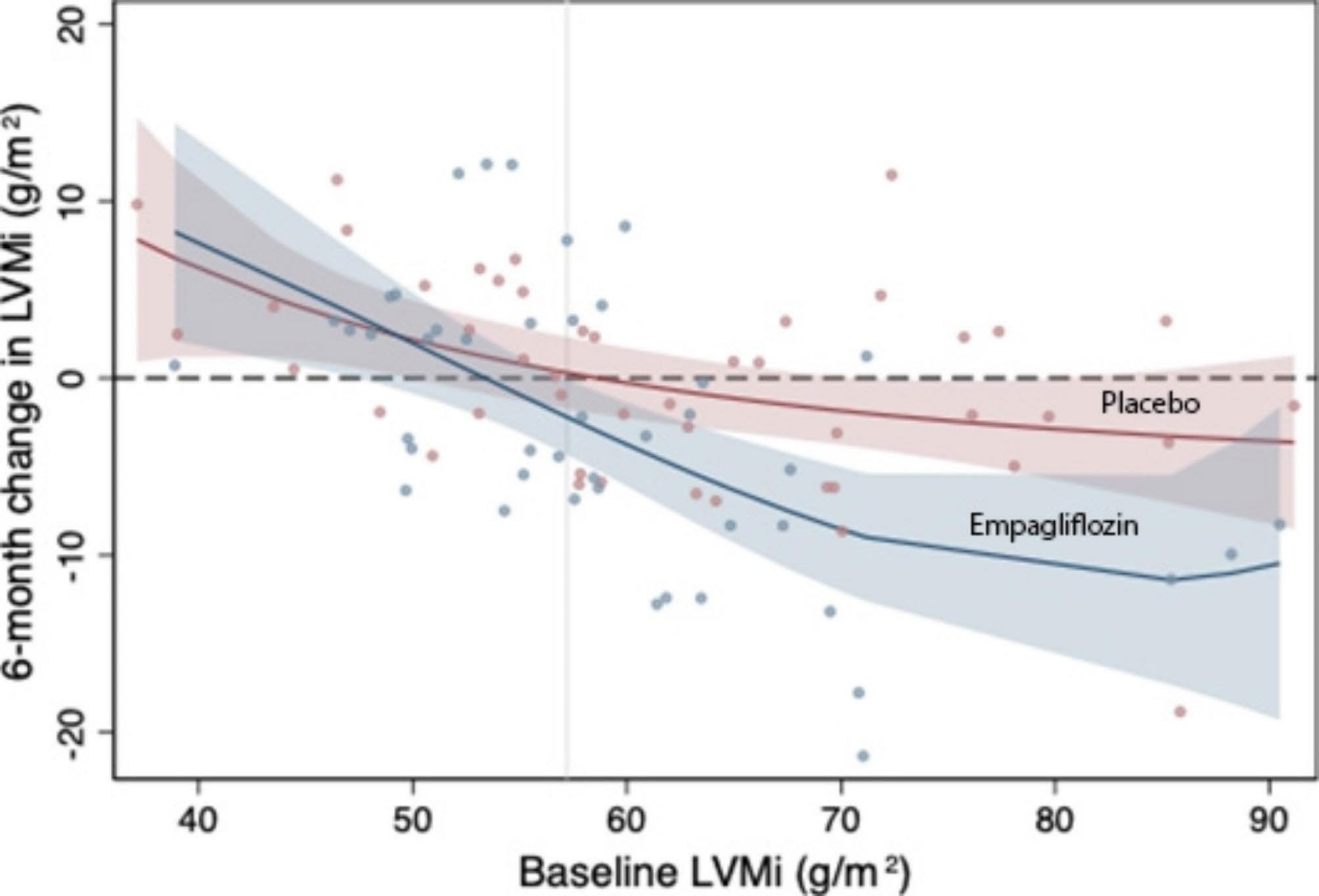
Treatment with empagliflozin (10 mg/d) and 6-month change in LVMi estimated over baseline LVMi values fitted to fractional-polynomial prediction with associated 95% confidence intervals. LVMi, left ventricular mass indexed to body surface area

The 6-month change in LVMi from estimation of a fractional polynomial of baseline LVMi stratified by the treatment arm is shown in Fig. 2 along with associated 95% CIs. A regression in LVMi was observed in patients with a baseline LVMi over 57.2 g/m^2^ when treated with empagliflozin and the degree of LVMi regression appeared to increase as baseline LVMi was increased, while no significant association between 6-month change in LVMi and baseline LVMi was found in the placebo group.

### Secondary outcomes

**Fig. 3:**
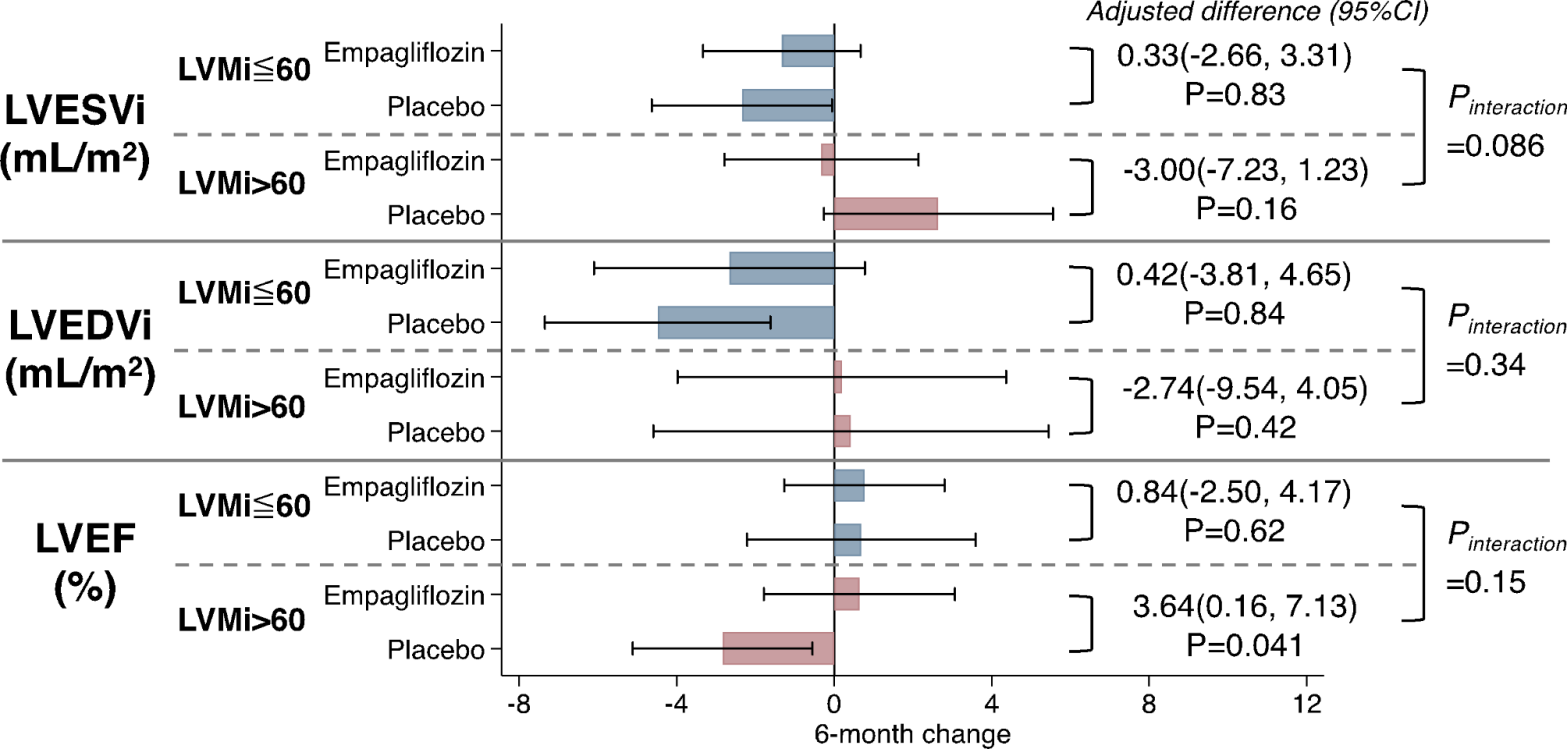
Association between treatment with empagliflozin (10 mg/d) and 6-month changes in LVESVi, LVEDVi, and LVEF stratified by baseline LVMi of ≤ 60 g/m^2^ or > 60 g/m^2^

In analyses evaluating the relationship of baseline LVMi and change in LVESVi from baseline to 6 months, we observed no significant association (*p*-for-interaction = 0.086; Fig. 3). Similarly, we found no association between baseline LVMi and change in LVEDVi (*p*-for-interaction = 0.34) nor between baseline LVMi and change in LVEF (*p*-for-interaction = 0.15). In addition, our analyses also demonstrated no significant association between baseline LVMi and baseline LVEDV, LVEDVi, LVESV, LVESVi, or LVEF (*p* > 0.05 for all; Table 2).

LVEDVi; left ventricular end diastolic volume indexed; LVESVi, left ventricular end systolic volume indexed; LVEF, left ventricular ejection fraction.

## Discussion

In this exploratory sub-analysis of the EMPA-HEART CardioLink-6 trial, we evaluated the influence of baseline LVMi on cardiac reverse remodelling with empagliflozin. Our analysis yielded the key finding that patients with a baseline LVMi > 60 g/m^2^ experienced significantly greater LVMi regression than those with baseline LVMi ≤ 60 g/m^2^ following 6-months of treatment with empagliflozin.

Reductions in LVM are associated with cardiovascular risk reduction and improved clinical outcomes [21, 22, 24]. Specifically, in the Heart Outcomes Prevention Evaluation (HOPE) trial investigating the angiotensin-converting enzyme inhibitor, ramipril, patients who experienced regression/prevention of LVH had significantly lower risk of cardiovascular death, myocardial infarction, or stroke (*p* < 0.0001) [21]. This highlights the important influence of LVM on clinical outcomes and supports the notion that the cardioprotective benefits associated with SGLT2i translate, at least in part, from LVM regression.

A post hoc subgroup analysis of the EMPA-REG OUTCOME trial demonstrated that risk reduction of 3-point MACE with empagliflozin was greater in patients who had LVH at baseline when compared those without [25]. These results align well with the findings of the current analysis which suggest that patients with a greater LVMi at baseline experienced greater LVM regression following treatment with empagliflozin than with a lower baseline LVMi.

In a recent meta-analysis of RCTs using cMRI to examine SGLT2i-mediated cardiac reverse remodelling in patients with T2D and/or heart failure, treatment with an SGLT2i was associated with a significant reduction in LVM [9]. When compared to the baseline LVMi values in the EMPA-HEART CardioLink-6 cohort of patients with T2D and CAD (SGLT2i arm = 59.3 g/m^2^ (± 10.9); placebo arm = 62.2 g/m^2^ (± 12.8)), other SGLT2i reverse remodelling trials that have recruited patients with heart failure with reduced ejection fraction have reported slightly higher baseline LVMi, though it must be noted that some of these trials used a slightly different measurement methodology for LVMi. Specifically, the baseline LVMi values in the SUGAR-DM-HF (Studies of Empagliflozin and Its Cardiovascular, Renal and Metabolic Effects in Patients With Diabetes Mellitus, or Prediabetes, and Heart Failure), EMPA-TROPISM (EMPA-TROPISM Trial: Are the “Cardiac Benefits” of Empagliflozin Independent of Its Hypoglycemic Activity?), and REFORM trials (Research Into the Effect of SGLT2 Inhibition on Left Ventricular Remodeling in Patients With Heart Failure and Diabetes Mellitus) were 61.2 g/m^2^ (± 16.1 g/m^2^), 67.9 g/m^2^ (± 17.8 g/m^2^), and 69.5 g/m^2^ (± 16.3 g/m^2^) in the SGLT2i arms and 65.4 g/m^2^ (± 19.6), 65.9 g/m^2^ (± 19.8), and 73.7 g/m^2^ (± 19.3) in the placebo arms, respectively [26–28]. Interestingly, despite enrolling patients with an average baseline LVMi that was similar to the LVMi > 60 g/m^2^ cohort in the current analysis (69.7 g/m^2^ (64.2–76.1)) and larger than in the other trials mentioned, the REFORM trial reported no significant regression in LVMi after 12 months of treatment with dapagliflozin (10 mg/d); the reasons for this are unclear and may be spurious in the setting of small sample sizes, variations in treatment duration, or differences in clinical characteristics of patient populations enrolled [26, 28]. The greatest LVMi regressions were observed in the EMPA-TROPISM trial which reported that treatment with empagliflozin (10 mg/d) for 6 months reduced LVMi by 8.5 g/m^2^ (± 15.9) [26]. Similar results suggesting reverse remodelling benefits of SGLT2i were observed in a meta-analysis of RCTs which assessed cardiac parameters using echocardiography as well as in another recent meta-analysis of observational studies [29, 30].

This study provides important and valuable information regarding the role of empagliflozin in cardiac reverse remodelling in patients with diabetes, however it must be noted that the current study also has limitations. First, the sample size was small. Second, the participants in this study were only followed for 6 months and it has been shown that, at least after an acute MI, LV remodelling can continue for as long as 2 years. Third, given the nature of this analysis, the findings should be considered hypothesis-generating.

## Conclusions

In conclusion, patients with larger LVMi at baseline experienced significantly greater cardiac reverse remodelling with empagliflozin than patients with a lower LVMi at baseline. Studies with larger cohorts and longer follow-ups are warranted to investigate the influence of baseline LVM on SGLT2i-mediated cardiac reverse remodelling and the treatment benefits received from treatment with empagliflozin.

## Data Availability

The datasets analyzed during the current study are not publicly available but are available from the corresponding author on reasonable request.

## List of Abbreviations

cMRI: Cardiac magnetic resonance imaging
CV: Cardiovascular
IQR: Interquartile ranges
LV: Left ventricular
LVEDV: Left ventricular end-diastolic volume
LVEDVi: Left ventricular end-diastolic volume indexed
LVEF: Left ventricular ejection fraction
LVESV: Left ventricular end-systolic volume
LVESVi: Left ventricular end-systolic volume indexed
LVH: Left ventricular hypertrophy
LVM: Left ventricular mass
LVMi: Left ventricular mass indexed
RCT: Randomized controlled trial
SD: Standard deviation
SGLT2i: Sodium-glucose transport protein 2 inhibitors
T2D: Type 2 diabetes
